# The death risk of pediatric patients with cancer-related sepsis requiring continuous renal replacement therapy: a retrospective cohort study

**DOI:** 10.1016/j.jped.2024.04.004

**Published:** 2024-05-24

**Authors:** Xiaoxuan Ma, Jiaying Dou, Chunxia Wang, Huijie Miao, Jingyi Shi, Yun Cui, Yiping Zhou, Yucai Zhang

**Affiliations:** a Department of Critical Care Medicine, Shanghai Children's Hospital, School of Medicine, Shanghai Jiao Tong University, Shanghai, China; b Institute of Pediatric Infection, Immunity, and Critical Care Medicine, Shanghai Children's Hospital, School of Medicine, Shanghai Jiao Tong University, Shanghai, China; c Institute of Pediatric Critical Care, Shanghai Jiao Tong University, Shanghai, China

**Keywords:** Sepsis, Cancer-related sepsis, Continuous renal replacement therapy, Mortality, PICU

## Abstract

**Objective:**

To assess the outcome of patients with cancer-related sepsis requiring continuous renal replacement therapy (CRRT) in a single-center pediatric intensive care unit (PICU).

**Method:**

Children with sepsis who necessitate CRRT from January 2017 to December 2021 were enrolled. The patients with leukemia/lymphoma or solid tumors were defined as underlying cancer. Multivariate logistic regression analysis was performed to identify the death risk factors in patients with cancer-related sepsis.

**Results:**

A total of 146 patients were qualified for inclusion. Forty-six (31.5%) patients with cancer-related sepsis and 100 (68.5%) non-cancer-related sepsis. The overall PICU mortality was 28.1% (41/146), and mortality was significantly higher in cancer-related sepsis patients compared with non-cancer patients (41.3% *vs*. 22.0%, *p* = 0.016). Need mechanical ventilation, p-SOFA, acute liver failure, higher fluid overload at CRRT initiation, hypoalbuminemia, and high inotropic support were associated with PICU mortality in cancer-related sepsis patients. Moreover, levels of IL-6, total bilirubin, creatinine, blood urea nitrogen, and international normalized ratio were significantly higher in non-survivors than survivors. In multivariate logistic regression analysis, pediatric sequential organ failure assessment (p-SOFA) score (OR:1.805 [95%CI: 1.047–3.113]) and serum albumin level (OR: 0.758 [95%CI: 0.581 -0.988]) were death risk factors in cancer-related sepsis receiving CRRT, and the AUC of combined index of p-SOFA and albumin was 0.852 (95% CI: 0.730–0.974).

**Conclusion:**

The overall PICU mortality is high in cancer-related sepsis necessitating CRRT. Higher p-SOFA and lower albumin were independent risk factors for PICU mortality.

## Introduction

With the tremendous advances of chemotherapy in patients with cancer or hematologic malignancies, there is an increasing number of patients admitted to the intensive care unit (ICU) due to cancer-related sepsis with potentially reversible complications.^1^^,^^2^ Sepsis and acute kidney injury (AKI) are the main complications in hemato-oncologic patients during hospitalization. These complications were associated with attributable mortality.^3^^,^^4^ Several studies have reported AKI in critically ill cancer patients,^5^^,^^6^ but fewer addressed the differences between cancer-related and non-cancer patients in the pediatric population.

Continuous renal replacement therapy (CRRT) has become widely used in critically ill children with AKI, fluid overload (FO), and electrolyte imbalances as it acts continuous and programmed removal of fluids as well as waste products.^7^^,^^8^ Raymakers-Janssen et al.^9^ reported the sad mortality (54.4%) of pediatric cancer and hematopoietic stem cell transplant patients requiring CRRT in the Netherlands. However, there is a limited study assessing the outcome in pediatric cancer or leukemia patients complicated with sepsis who received CRRT. A better understanding of the need for CRRT in these patients that influences outcomes is essential for optimal therapy in cancer patients in PICU.

The aim of this study was to assess the outcome and risk factors of patients with cancer/ leukemia complicated by sepsis who required CRRT in a single-center PICU.

## Methods

### Study patient cohorts

In this retrospective analysis, patients with sepsis who received CRRT due to AKI or FO (aged from 28 days to 18 years old) admitted to PICU were selected as a cohort from Jan. 2017 to Dec. 2021. Patients with solid malignancies or leukemia were categorized in “cancer-related sepsis” group, and those without malignancies were categorized in “non- cancer-related sepsis” group. The exclusion criteria were: (1) the patient receipt of liver transplant in the preceding 60 days before initial CRRT; (2) the patients receiving hematopoietic stem cell transplantation; (3) congenital immunodeficiency diseases; (4) patients who received immunosuppressant therapy over 14 days before CRRT (defined as receiving methylprednisolone ≥ 2 mg/kg.d or equivalent dose of steroid ≥ 14 days within 7 days before initial CRRT); (5) patients who were stayed less than 24 h in PICU.

Pediatric severe sepsis and sepsis-associated organ dysfunction were defined according to the 2005 International Pediatric Sepsis Consensus Conference criteria^10^ and International Classification of Diseases 9th Edition codes (ICD 9). AKI was defined and classified according to the Kidney Disease: Improving Global Outcomes (KDIGO) criteria, which include incremental changes in serum creatinine (sCr) and decremental urine output.^11^ FO was calculated as the body weight greater percent,%FO = [Fluid in-Fluid out] (L)/PICU admission weight (kg) × 100%.^12^ Indications for initiation of CRRT are FO and AKI in the PICU. The vasoactive inotropic score (VIS) was calculated as ([epinephrine+ norepinephrine] μg/ kg.min) × 100 + (dobutamine + dopamine] μg/kg.min) + [milrinone μg/kg.min] × 10 + [vesopressin μg/kg.min]× 10000.^13^

### Ethical considerations

The ethics committee of Shanghai Children's Hospital, Shanghai Jiao Tong University School of Medicine (2020RY047-E01) approved the study. Because no additional interventions were performed, the informed consent was waived by the institutional review board, and all data used were anonymized and didn't be traced back to individual patients.

### Data collection

Baseline characteristics of PICU admission and treatment were obtained from hospital medical records. The severity of patients was assessed by PRISM III score ^14^ and pediatric sequential organ failure assessment (p-SOFA) score.^15^ Oncology diagnosis, type of PICU admission, the origin of infection, and reason for PICU admission were collected. FO at CRRT initiation was calculated. The modality of respiratory support at initial CRRT, interval time between PICU admission and CRRT initiation, vasoactive support and VIS value at CRRT initiation, and total duration of CRRT were recorded.

Laboratory variables, indicators of inflammatory response including white blood cell count (WBC), platelet count (PLT), C-reactive protein (CRP), Procalcitonin (PCT), interleukin-6 (IL-6), and IL-8 were collected at CRRT initiation. The other variables including lactate, PaO_2_/FiO_2_, PaCO_2_, fibrinogen, international normalized ratio (INR), prothrombin time (PT), D-dimer, potassium, total bilirubin, alanine aminotransferase (ALT), blood urea nitrogen (BUN), serum creatinine (sCr), total protein, and albumin at CRRT initiation were extracted from medical records. The primary outcome was PICU mortality. Other outcomes were the length of PICU stay and the risk factors related to the death.

### Statistical analysis

Data analyses were performed by SPSS 22.0 software (IBM, Armonk, NY, USA). Continuous variables were appropriately expressed as mean ± standard deviation (mean ± SD) or median (interquartile range, IQR) and analyzed using the Student's *t*-test or Mann-Whitney *U* test, respectively. The *chi*-squared test was used to compare the categorical data. Odds ratios (*ORs*) and 95% confidence intervals (*CI*) were calculated using multivariate *logistic* regression analyses. To assess the capacity of p-SOFA and albumin to act as predictors of PICU mortality, a receiver operating characteristic (ROC) curve was generated, and the area under the ROC curve (AUC) and 95% *CI* were shown. All statistical tests were two-tailed and a value of *p* < 0.05 was considered statistically significant.

## Result

### Study population

**Figure 1:**
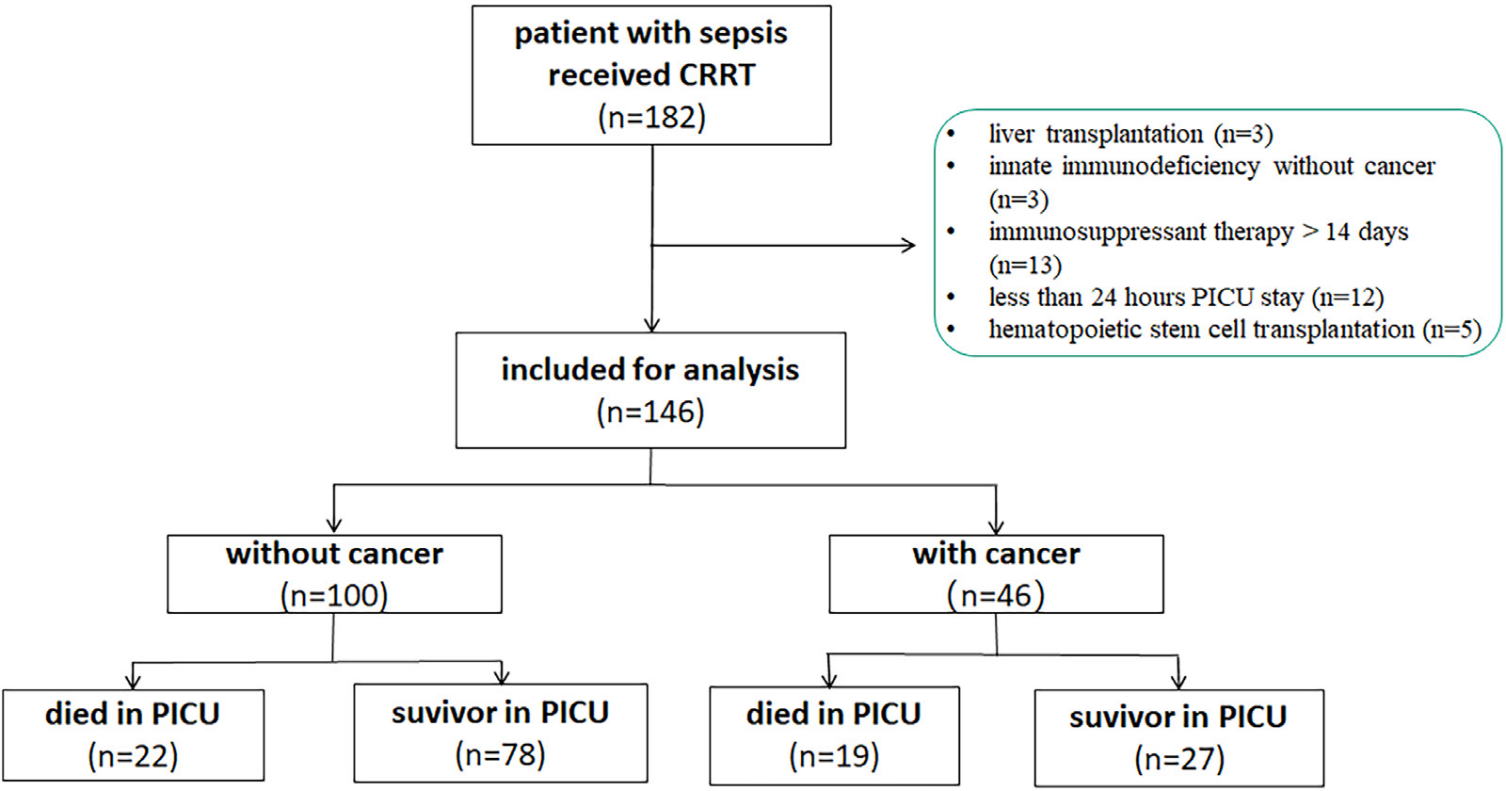
Study flowchart. CRRT: continuous renal replacement therapy; PICU: pediatric intensive care unit.

During the study period, there were 7083 patients admitted to the PICU, of which 236 admissions had an underlying malignancy or solid cancer. A total of 182 patients with severe sepsis who received CRRT were enrolled. Among them, 36 patients were excluded including 3 cases of liver transplantation, 12 cases stayed less than 24 h in PICU, 5 patients received hematopoietic stem cell transplantation, 3 cases of innate immunodeficiency, and 13 patients received immunosuppressant therapy over 14 days before CRRT. Finally, 146 patients with severe sepsis who received CRRT were analyzed (Figure 1).

### Baseline characteristics

There were no significant differences between the cancer-related sepsis group and the non-cancer-related sepsis group regarding the origin of infection and reason for PICU admission. Patients were predominantly male (55.5%), but there were significant differences in aspect of age (84 [45.75,130.25] *vs.*34 [14.25, 85.25], *p* < 0.001) months, PRISM III score (7.5 *vs.* 13, *p* < 0.001), and p-SOFA score (11^,^ *vs.* 6, *p* < 0.001) between cancer-related sepsis group and non-cancer-related sepsis group. The proportion of bacterial+fungal infection in cancer-related sepsis group was significantly higher than that in non-cancer-related sepsis group (22.0% *vs.* 5.0%, *p* = 0.005) (Supplement 1).

### Outcomes of patients with cancer-related sepsis received CRRT

The overall PICU mortality of patients with sepsis requiring CRRT was 28.1% (41/146 patients). The mortality was significantly higher in cancer-related sepsis compared with non-cancer patients (41.3% *vs.* 22.0%, *p* = 0.016) (Supplement 1).

### Clinical characteristics at CRRT initiation in patients with cancer-related sepsis received CRRT

Table 1 summarizes the baseline characteristics at CRRT initiation of survivors and non-survivors in the group of patients with cancer-related sepsis. FO (189 [145, 267] ml/kg *vs.* 131 [118, 276] ml/kg, *p* = 0.011), percentage of ventilator respiratory support (100% *vs.*70.4%, *p* = 0.014), and VIS (140 [35, 300] *vs.*55 [10, 90], *p* = 0.043) were higher in non-survivors than survivors. Higher p-SOFA and lower albumin were displayed in non-survivors than survivors. In addition, there were 4 cases complicated by acute liver failure in 19 non-survivors, and there was no case of acute liver failure in survivors.

**Table 1:** Variables in patients with cancer-related sepsis required CRRT.

variables	Total patients (*n* = 46)	Survivors (*n* = 27)	non-survivors (*n* = 19)	*X^2^ or Z*	*P value*
Oncology diagnosis, n (%)					
Leukemia	38 (82.6)	24 (88.9)	14 (73.7)	/	0.246
lymphoma	2 (4.3)	0	2 (10.5)	/	0.165
Solid tumor	6 (13.0)	3 (11.1)	3 (15.8)	/	0.680
p-SOFA score	11 (8,13)	9 (8,12)	13 (12,14)	−3.638	<0.001*
BMI, median (IQR), kg/m^2^	16 (14,18)	16 (14,18)	16 (14,18)	−0.428	0.668
Origin of infection, n (%)					
Respiratory	18 (39.1)	12 (44.4)	6 (31.6)	0.775	0.379
Gastrointestinal tract	14 (30.4)	9 (33.3)	5 (26.3)	0.259	0.611
Blood flow	13 (28.3)	6 (22.2)	7 (36.8)	1.176	0.278
Other	1 (2.2)	0	1 (5.3)	/	0.413
Reason of PICU admission					
shock	33	20	13	0.176	0.675
acute kidney injury	8	3	5	/	0.246
Respiratory failure	34	17	17	2.806	0.094
Acute liver failure	4	0	4	/	0.024*
Gastrointestinal dysfunction	21	11	10	0.636	0.425
Encephalopathy	6	1	5	/	0.068
MODS	18	6	12	7.846	0.005*
Fluid overload at CRRT initiation, median (IQR), ml/kg	150 (120.8, 230)	131 (118,276)	189 (145,267)	−2.533	0.011*
0% to ≤ 10%, n	6	4	2	/	>0.999
10% to ≤ 20%, n	26	18	8	2.738	0.098
> 20%, n	14	5	9	4.384	0.036*
Modality of respiratory support at initial CRRT, n (%)					
Ventilator	38 (82.6)	19 (70.4)	19 (100)	/	0.014*
High flow nasal oxygen	3 (6.5)	3 (11.1)	0	/	0.257
Mask	5 (10.9)	5 (18.5)	0	/	0.067
Vasoactive support at CRRT initiation, n (%)	41 (89.1)	24 (58.5)	17 (41.5)	/	>0.999
VIS, median (IQR)	60 (25.0, 157.5)	55 (10,90)	140 (35,300)	−2.023	0.043*
Admitted PICU to first CRRT, h, median (IQR)	23.5 (12.8,58.5)	29 (11,56)	20 (15,68)	−0.145	0.885
Total duration of CRRT, h, median (IQR)	37.5 (20.8,71.0)	35 (22,71)	43 (17,71)	−0.145	0.885
Bleeding related to CRRT, n (%)	7 (15.2)	2 (7.4)	5 (26.3)	3.090	0.079
Length of PICU stay, d, median (IQR)	9 (6,14)	10 (8,15)	7 (3,10)	−1.757	0.079

p-SOFA, pediatric sequential organ failure assessment; PICU, pediatric intensive care unit; CNS, center nervous system; CRRT, continuous renal replacement therapy; VIS, vasoactive inotropic score; MODS, multiple organ dysfunction syndrome.

### Laboratory variable at CRRT initiation in patients with cancer-related sepsis received CRRT

As shown in Table 2, there were significant differences regarding the values of laboratory indexes at CRRT initiation between non-survivors and survivors in the cancer-related sepsis group. Importantly, p-SOFA scores (13 *vs.* 9, *p* < 0.001) and INR (1.55 *vs.*1.43, *p* = 0.035) were significantly higher in non-survivors than survivors (Supplement 2 A, B). In addition, serum levels of IL-6, total bilirubin, BUN, and sCr were higher in non-survivors than survivors (2724.6 pg/ml*vs.* 289.2 pg/ml, *p* = 0.042; 47 μmol/L *vs.* 22 μmol/L, *p* = 0.006; 9.1 mg/dL *vs.* 5.2 mg/dL, *p* = 0.001; 62 μmol/L *vs.* 32 μmol/L, *p* = 0.008) (Supplement 2 C-F). However, serum levels of albumin were obviously lower in non-survivors than survivors (28.3 g/L *vs.* 32.8 g/L, *p* = 0.014) (Supplement 2 G).

**Table 2:** Laboratory variables at CRRT initiation in patients with cancer-related sepsis required CRRT (IQR).

variables	Total patients (*n* = 46)	Survivors (*n* = 27)	non-survivors (*n* = 19)	*X^2^ or Z*	*P value*
WBC count(10^9^/L)	0.44 (0.10,1.54)	0.46 (0.16,1.30)	0.12 (0.06,2.28)	−0.882	0.378
platelet count (10^9^/L)	24 (7.75,88.75)	33 (15,92)	15 (7,26)	−1.674	0.094
CRP, mg/L	141 (78,160)	120 (49,157)	150 (100,160)	−1.240	0.215
Procalcitonin, ng/ml	8.31 (0.84,20.81)	4.13 (0.95,21.27)	15 (7,26)	−0.033	0.973
IL-6, pg/ml	595.25 (145.90,4976.99)	289.2 (73.4,4018.5)	2724.6 (551.3,7063.9)	−2.030	0.042*
IL-8, pg/ml	733.91 (15.82,3169.26)	143.38 (1.74,2137.67)	1207.13 (475.72,4066.60)	−1.757	0.079
Lactate, mmol/L	3.6 (2.2,5.75)	3 (2.1,4.9)	4.2 (3.0,8.3)	−1.719	0.086
PaO_2_/FiO_2_, mmHg	217.2 (97.8,303.4)	245 (144.9,325.0)	192 (89.3,260.6)	−1.652	0.098
PaCO_2_, mmHg	5.2 (4.65,6.20)	5.02 (4.40,5.60)	5.5 (4.7,7.2)	−1.317	0.188
Total bilirubin, µmol/L	27.5 (10.4,46.9)	22 (9.2,33.5)	47 (19.5,93.3)	−2.766	0.006*
ALT, IU	31.5 (17.75,58.50)	28 (19,46)	42 (14,109)	−0.893	0.372
BUN, mg/dL,	6.55 (3.88,10.50)	5.2 (2.7,8.2)	9.1 (6.4,16.1)	−3.313	0.001*
sCr, µmol/L	38.5 (26.75, 88.75)	32 (23,49)	62 (36,130)	−2.634	0.008*
Total protein, g/L	54.03 (50.78,57.38)	53.1 (50.4,56.02)	55.52 (51.10,59.10)	−0.948	0.343
Albumin, g/L	31.94 (26.78,35.97)	32.8 (29.4,36.51)	28.3 (24.25,32.50)	−2.465	0.014
≥35 g/L, n (%)	19 (41.3)	13 (48.1)	6 (31.6)	1.263	0.261
< 35 g/L, n (%)	27 (58.7)	14 (51.9)	13 (68.4)	1.263	0.261
Fibrinogen (g/L)	2.45 (1.47,3.01)	2.66 (1.32,3.16)	2.27 (1.60,2.95)	−0.517	0.605
INR	1.52 (1.31,1.72)	1.43 (1.20,1.60)	1.55 (1.39,1.82)	−2.103	0.035*
PT, s	16.1 (14.0,19.20)	15.85 (13.9,18.6)	16.20 (14,19.8)	−0.494	0.621
D-dimer, mg/L	3.6 (1.8,10.4)	2.6 (1.8,6.2)	5.1 (2.6,16.7)	−1.574	0.115

WBC, White blood cell; CRP, C-reactive protein; IL-6, interleukin-6; IL-8, interleukin-8; PaO_2_/FiO_2_, ratio of PaO_2_ to the fraction of inspired oxygen; ALT, alanine aminotransferase; BUN, blood urea nitrogen; sCr, serum creatinine; INR, International Normalized Ratio; PT, Prothrombin time.

### Multivariate logistic regression analysis of risk factors and ROC analysis for PICU mortality

**Figure 2:**
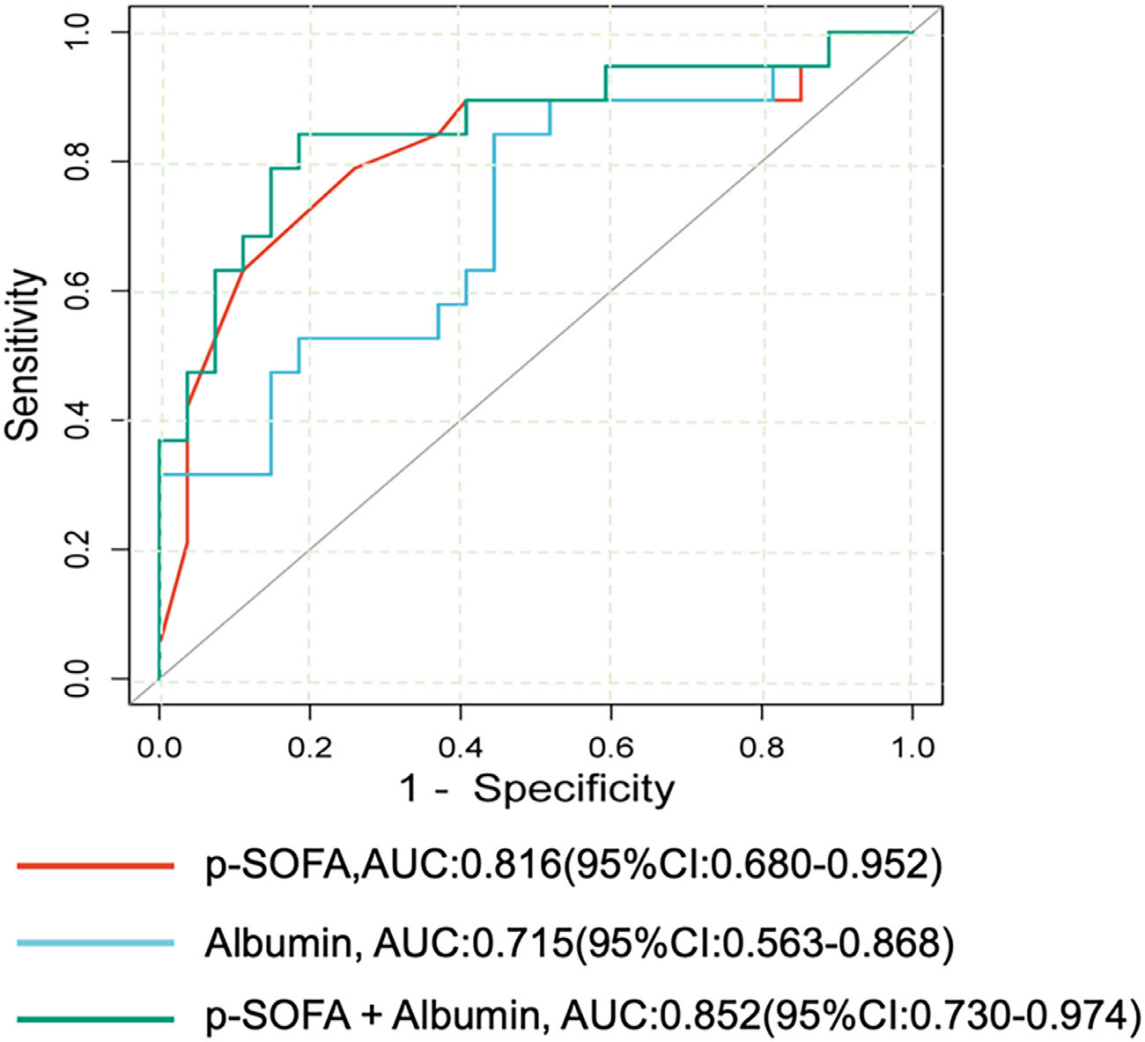
ROC curves analyses of p-SOFA score, albumin, and combined index of both for predicting the PICU mortality.

Multivariate *logistic* regression analysis indicated that higher p-SOFA score (*OR*: 1.805 [95% *CI*:1.047,3.113]) and low albumin level (*OR*: 0.758[95% *CI*: 0.581,0.988]) were independently associated with increased death risk (Supplement 3). The area under the ROC curve (AUC) of p-SOFA score and albumin for predicting PICU mortality is shown in Figure 2. Among them, the AUC of the p-SOFA score was 0.816(95% *CI*: 0.680 - 0.952) with a sensitivity of 78.9% and a specificity of 74.1% at the cutoff value of 11.5. In addition, the AUC of albumin was 0.715 (95% *CI*: 0.563- 0.868) with a sensitivity of 84.2% and a specificity of 55.6% at the cutoff value of 32.6 g/L. Moreover, the AUC of the combination of p-SOFA and albumin levels for predicting the PICU mortality was 0.852(95%*CI:* 0.730–0.974) with a sensitivity of 73.7%, specificity of 88.9% at the cutoff value of 0.48, which obtained a trend superior to the predictive capacity of albumin (*p* = 0.000) alone.

## Discussion

Accompanying septic AKI or FO is an ominous sign in patients with solid malignancies/leukemia. In the retrospective analysis, PICU mortality in patients with cancer-related sepsis required CRRT was 41.3%, which was significantly higher than that in patients with non-cancer-related sepsis received CRRT (*p* = 0.016). With regard to PICU mortality in patients with cancer-related sepsis, need mechanical ventilation, acute liver failure, higher IL-6 level, hypoalbuminemia, and higher inotropic support were associated with a higher PICU mortality. The authors found that patients with higher p-SOFA scores and lower blood albumin levels at CRRT initiation were independent death risk factors.

Certainly, sepsis is more involved in immunocompromised patients with cancer/leukemia.^16^ The question of whether cancer with sepsis contributed to additional risk remains non-well answered. Hensley et al. ^17^ reported in-hospital mortality in cancer-related-sepsis was 27.9% *versus* 19.5% in non-cancer-related sepsis based on the US National Readmissions Database (2013–2014). The mortality difference was greatest in younger patients and waned with age. In 1927 PICU admissions of pediatric cancer and hematopoietic stem cell transplant patients of 8 PICU in the Netherlands, 68 patients required CRRT and PICU mortality was 54.4%. Fluid overload (*OR:*1.08; 95% *CI*: 1.01–1.17) and the need for inotropic support (*OR:* 6.53[95% *CI*: 1.86–23.08]) at the start of CRRT were associated with mortality.^9^ In the previous study, the in-hospital mortality of patients with cancer-related sepsis or with non-cancer-related sepsis were 36.3% (49/135) and 9.3% (59/633), respectively (*p* < 0.01).^18^ In the present cohort analysis, a total of 146 patients with severe sepsis received CRRT. Of whom, there were 46 (31.5%) patients with cancer-related sepsis and 100 (68.5%) cases with non-cancer-related sepsis, and PICU mortality was significantly higher in cancer-related sepsis patients compared with non-cancer patients (41.3% *vs*. 22.0%, *p* = 0.016).

The authors identified two important risk factors for PICU mortality: higher p-SOFA score and lower albumin level at CRRT initiation. Generally accepted risk factors for AKI or FO were sepsis and septic shock in PICU.^19^ Patients who were more critically ill or more hemodynamically unstable usually present FO. However, the authors did not find a significant association between the FO and PICU mortality in the present study. The possible reason might be the timely removal of excess fluid by CRRT. The p-SOFA score was developed by adapting the original SOFA score as the scoring system to quantify organ dysfunction in the Third International Consensus Definitions for Sepsis and Septic Shock (Sepsis-3).^15^^,^^20^ In the present study, the authors found that the p-SOFA score was significantly higher in non-survivors than in survivors (*p*<0.001), and higher p-SOFA score and low albumin level were independently associated with increased death risk of cancer-related septic children. Multiple studies have shown that the p-SOFA score had discrimination for hospital mortality in patients with suspected infection in the emergency department ^21^ and critically ill children,^15^ and utilizing the p-SOFA score cutoff of 8 and an increase in ΔSOFA (day 3 and day 1) of > 2 have a greater discriminative power for predicting in-hospital mortality than either PRISM III score or PELOD-2 score.^22^ In the present study, the AUC of the p-SOFA score as a prognostic factor was 0.816 (95% *CI*: 0.680 - 0.952) with a sensitivity of 78.9% and a specificity of 74.1% at the cutoff value of 11.5. This is the first report about p-SOFA as an additional discrimination factor for hospital mortality in cancer-related septic children. Moreover, accumulated evidence indicated that hypoalbuminemia is an important indicator of extensive capillary leakage syndrome induced by sepsis or septic shock, as well as a risk factor for poor prognosis.^23^^,^^24^ As a second-line and adjunctive to crystalloids for fluid resuscitation in hypovolemic shock, sepsis and septic shock, albumin clinical use is supported with a low to moderate quality of evidence.^25^ More importantly, albumin is critical for the intact innate and adaptive immune responses depending on the interaction of albumin with bioactive lipid mediators that play an important role in antimicrobial defense.^26^ The recent study reported that sepsis-associated hypoalbuminemia was mainly caused by enhanced clearance from the circulation.^27^ So, the authors suspected that there could be multiple contributors to the final feature of low albumin level, but this point should be paid more attention to in cancer-related septic children. The previous study based on the MIMIC Ⅲ database indicated that the use of vasopressor, INR ≥ 1.5, and quick SOFA (qSOFA) score are associated with hospital mortality in patients with sepsis who received CRRT.^28^ So, hypoalbuminemia could be an alternative prognostic factor for cancer-related septic children under CRRT, like use of vasopressor and INR in critically ill adults undergoing CRRT.

The present study has some limitations. Firstly, the present study was conducted in a single PICU. Therefore, the result of this study could not be generalized to general hematology & oncology. Secondly, the matched baseline was not performed between cancer-related and non-cancer-related sepsis due to the small sample size. Thirdly, given the relatively short interval time between PICU admission and CRRT initiation in the present study, the value of p-SOFA, VIS, PRISM III score was not determined at each time point throughout the sepsis. Fourthly, the prevalence of multi-drug resistant (MDR) bacteria sepsis which may carry a worse prognosis was lacking in the present study. Fifthly, immunosuppressant therapy leads to potential adverse side effects and offers the patients tolerating the increased risk of hyperglycemia, catabolism-related diffuse neuromuscular weakness (including the diaphragm), and hospital-acquired infections.^29^ However, this result showed that the predictive capacity of a combined index of p-SOFA and albumin was with an AUC of 0.854 (95%*CI*: 0.734–0.973). These results will give an insight for assessing the prognosis in patients with cancer-related patients who would need CRRT support.

In this retrospective cohort analysis, the authors found that PICU mortality was high in pediatric cancer-related sepsis requiring CRRT. Higher p-SOFA scores and lower blood albumin levels at CRRT initiation were independent death risk factors for predicting mortality in PICU.

## Conflicts of interest

The authors declare no potential conflicts of interest with respect to the study, authorship, and/or publication of this article. The authors emphasize that none of the corporations were involved in the laboratory method or sponsoring the study.

## Appendix Supplementary materials

Image, application 1

## References

[1] Díaz-Díaz D., Villanova Martínez M., Palencia Herrejón E. (2018). Oncological patients admitted to an intensive care unit. Analysis of predictors of in-hospital mortality. Med Intensiva (Engl Ed).

[2] Zampieri F.G., Romano T.G., Salluh J.I., Taniguchi L.U., Mendes P.V., Nassar A.P. (2021). Trends in clinical profiles, organ support use and outcomes of patients with cancer requiring unplanned ICU admission: a multicenter cohort study. Intensive Care Med.

[3] Awad W.B., Nazer L., Elfarr S., Abdullah M., Hawari F. (2021). A 12-year study evaluating the outcomes and predictors of mortality in critically ill cancer patients admitted with septic shock. BMC Cancer.

[4] Prasertsan P., Anuntaseree W., Ruangnapa K., Saelim K., Geater A. (2019). Severity and mortality predictors of pediatric acute respiratory distress syndrome according to the pediatric acute lung injury consensus conference definition. Pediatr Crit Care Med.

[5] Seylanova N., Crichton S., Zhang J., Fisher R., Ostermann M. (2020). Acute kidney injury in critically ill cancer patients is associated with mortality: a retrospective analysis. PLoS One.

[6] Martos-Benítez F.D., Soto-García A., Gutiérrez-Noyola A. (2018). Clinical characteristics and outcomes of cancer patients requiring intensive care unit admission: a prospective study. J Cancer Res Clin Oncol.

[7] Pekkucuksen N.T., Akcan Arikan A., Swartz S.J., Srivaths P., Angelo J.R (2020). Characteristics and clinical outcomes of prolonged continuous renal replacement therapy in critically ill pediatric patients. Pediatr Crit Care Med.

[8] Buccione E., Guzzi F., Colosimo D., Tedesco B., Romagnoli S., Ricci Z. (2021). Continuous renal replacement therapy in critically ill children in the pediatric intensive care unit: a retrospective analysis of real-life prescriptions. Complications, and Outcomes. Front Pediatr..

[9] Raymakers-Janssen P.A., Lilien M.R., Tibboel D., Kneyber M.C., Dijkstra S., van Woensel J.B. (2019). Epidemiology and outcome of critically ill pediatric cancer and hematopoietic stem cell transplant patients requiring continuous renal replacement therapy: a retrospective nationwide cohort study. Crit Care Med.

[10] Goldstein B., Giroir B., Randolph A. (2005). International consensus conference on pediatric Sepsis. International pediatric sepsis consensus conference: definitions for sepsis and organ dysfunction in pediatrics. Pediatr Crit Care Med..

[11] Khwaja A. (2012). KDIGO clinical practice guidelines for acute kidney injury. Nephron Clin Pract.

[12] Bhaskar P., Dhar A.V., Thompson M., Quigley R., Modem V. (2015). Early fluid accumulation in children with shock and ICU mortality: a matched case-control study. Intensive Care Med.

[13] Gaies M.G., Gurney J.G., Yen A.H., Napoli M.L., Gajarski R.J., Ohye R.G. (2010). Vasoactive-inotropic score as a predictor of morbidity and mortality in infants after cardiopulmonary bypass. Pediatr Crit Care Med.

[14] Gonçalves J.P., Severo M., Rocha C., Jardim J., Mota T., Ribeiro A. (2015). Performance of PRISM III and PELOD-2 scores in a pediatric intensive care unit. Eur J Pediatr.

[15] Matics T.J., Sanchez-Pinto L.N. (2017). Adaptation and validation of a pediatric sequential organ failure assessment score and evaluation of the sepsis-3 definitions in critically ill children. JAMA Pediatr.

[16] Wösten-van Asperen R.M., van Gestel J.P., van Grotel M., Tschiedel E., Dohna-Schwake C., Valla F.V. (2019). PICU mortality of children with cancer admitted to pediatric intensive care unit a systematic review and meta-analysis. Crit Rev Oncol Hematol.

[17] Hensley M.K., Donnelly J.P., Carlton E.F., Prescott H.C (2019). Epidemiology and outcomes of cancer-related versus non-cancer-related sepsis hospitalizations. Crit Care Med.

[18] Zhou T.L., Zhou Y.P., Zhang Y.C., Cui Y., Wang F., Chen R.X. (2020). Clinical features and outcomes of cancer-related *versus* non-cancer-related sepsis in pediatric intensive care unit. Zhonghua Er Ke Za Zhi.

[19] Stanski N.L., Cvijanovich N.Z., Fitzgerald J.C., Bigham M.T., Wong H.R. (2020). Genomics of pediatric septic shock investigators. Severe acute kidney injury is independently associated with mortality in children with septic shock. Intensive Care Med.

[20] Singer M., Deutschman C.S., Seymour C.W., Shankar-Hari M., Annane D., Bauer M. (2016). The third international consensus definitions for sepsis and septic shock (sepsis-3). JAMA.

[21] Balamuth F., Scott H.F., Weiss S.L., Webb M., Chamberlain J.M., Bajaj L. (2022). Validation of the pediatric sequential organ failure assessment score and evaluation of third international consensus definitions for sepsis and septic shock definitions in the pediatric emergency department. JAMA Pediatr.

[22] Lalitha A.V., Satish J.K., Reddy M., Ghosh S., George J., Pujari C (2021). Sequential organ failure assessment score as a predictor of outcome in sepsis in pediatric intensive care unit. J Pediatr Intensive Care.

[23] Druey K.M., Greipp P.R. (2010). Narrative review: the systemic capillary leak syndrome. Ann Intern Med.

[24] Patel A., Laffan M.A., Waheed U., Brett S.J. (2014). Randomised trials of human albumin for adults with sepsis: systematic review and meta-analysis with trial sequential analysis of all-cause mortality. BMJ.

[25] Abedi F., Zarei B., Elyasi S. (2024). Albumin: a comprehensive review and practical guideline for clinical use. Eur J Clin Pharmacol.

[26] Wiedermann C.J. (2021). Hypoalbuminemia as surrogate and culprit of infections. Int J Mol Sci.

[27] Omiya K., Sato H., Sato T., Wykes L., Hong M., Hatzakorzian R., Kristof A.S. (2021). Albumin and fibrinogen kinetics in sepsis: a prospective observational study. Crit Care.

[28] Wang C., Zheng J., Wang J., Zou L., Zhang Y. (2022). *Cox-LASSO* analysis for hospital mortality in patients with sepsis received continuous renal replacement therapy: a MIMIC-III database study. Front Med (Lausanne).

[29] Weiss S.L., Peters M.J., Alhazzani W., Agus M.S., Flori H.R., Inwald D.P. (2020). Surviving sepsis campaign international guidelines for the management of septic shock and sepsis-associated organ dysfunction in children. Pediatr Crit Care Med.

