# Supplementary material for: The death risk of pediatric patients with cancer-related sepsis requiring continuous renal replacement therapy: a retrospective cohort study
Source: J Pediatr (Rio J). 2024 May 24;100(6):614–21. doi: 10.1016/j.jped.2024.04.004 (PMC11662743; doi:10.1016/j.jped.2024.04.004)
Supplement: Supplementary file 1 [file mmc1.docx]

**JPED-D-23-00517_Supplementary Material**

**Supplement 1** Baseline Characteristics of Patients with or without cancer-related sepsis.

| Variables | Total (n=146) | cancer-related sepsis  (n=46) | Non-cancer-related sepsis (n=100) | *X^2^ or Z* | *P value* |
| --- | --- | --- | --- | --- | --- |
| Age, mo, median (IQR) | 47.5(19,97.5) | 84(45.8,130.3) | 34(14.3,85.3) | -4.173 | <0.001* |
| Male gender, n (%) | 81(55.5) | 27(58.7) | 54(54.0) | 0.281 | 0.596 |
| BMI, median (IQR), kg/m^2^ | 16(14,18) | 16(14,18) | 16.5(14,18) | -0.598 | 0.550 |
| PRSM III score, median (IQR) | 8(6,12) | 13(8,15) | 7.5(6,9) | -6.070 | <0.001* |
| p-SOFA score, median (IQR) | 8(5,11) | 11(8,13) | 6(5,9) | -5.317 | <0.001* |
| Type of PICU admission, n (%) |  |  |  |  |  |
| Emergency | 62(42.5) | 12(26.1) | 50(50.0) | 7.347 | 0.007* |
| Transferred | 84(57.5) | 34(73.9) | 50(50.0) | 7.347 | 0.007* |
| Origin of infection, n |  |  |  |  |  |
| Respiratory | 72 | 18 | 54 | 2.787 | 0.095 |
| Gastrointestinal tract | 37 | 14 | 23 | 0.920 | 0.337 |
| Blood flow | 27 | 13 | 14 | 4.250 | 0.039* |
| CNS | 5 | 0 | 5 | / | 0.326 |
| Other^★^ | 5 | 1 | 4 | / | >0.999 |
| Microbe species, n (%) |  |  |  |  |  |
| Bacteria | 82(56.0) | 27(59.0) | 55(55.0) | 0.175 | 0.676 |
| Bacteria+Fungi | 15(10.0) | 10(22.0) | 5(5.0) | 7.847 | 0.005* |
| Others* | 17(12.0) | 3(6.0) | 14(14.0) | 1.713 | 0.191 |
| Unknown | 32(22.0) | 6(13.0) | 26(26.0) | 3.091 | 0.079 |
| Reason for PICU admission |  |  |  |  |  |
| Shock | 107 | 33 | 74 | 0.082 | 0.774 |
| Acute kidney injury | 25 | 8 | 17 | 0.003 | 0.954 |
| Respiratory failure | 118 | 34 | 84 | 2.068 | 0.150 |
| Acute liver failure | 8 | 4 | 4 | 1.341 | 0.247 |
| Gastrointestinal dysfunction | 58 | 21 | 37 | 0.985 | 0.321 |
| Encephalopathy | 30 | 6 | 24 | 2.317 | 0.128 |
| Duration of CRRT, h, median (IQR) | 40(21,69) | 37.5(20.75,71.0) | 41(21,69) | -0.097 | 0.923 |
| Length of PICU stay, d, median (IQR) | 11(8,18) | 12(8.75,15.25) | 11(8,18.75) | -0.215 | 0.830 |
| PICU mortality, n (%) | 41(28.1) | 19(41.3) | 22(22.0) | 5.814 | 0.016* |

Other★ included skin and subcutaneous tissue infections 3 cases, urinary tract infections 2 cases.

p-SOFA: pediatric sequential organ failure assessment; PICU: pediatric intensive care unit; CNS: center nervous system; CRRT: continuous renal replacement therapy

Microbe species: ①Bacteria mainly included Streptococcus pneumoniae 21 cases, Escherichia coli 14, Acinetobacter baumannii 13 cases, Klebsiella pneumoniae 9 cases, Staphylococcus aureus 6 cases, etc. ②Fungi included Candida albicans 10cases, non-Candida albicans 2cases, and aspergillus 3cases. ③Others* included adenovirus 12cases, influenza virus 2 cases, and mycoplasma pneumoniae 3cases.


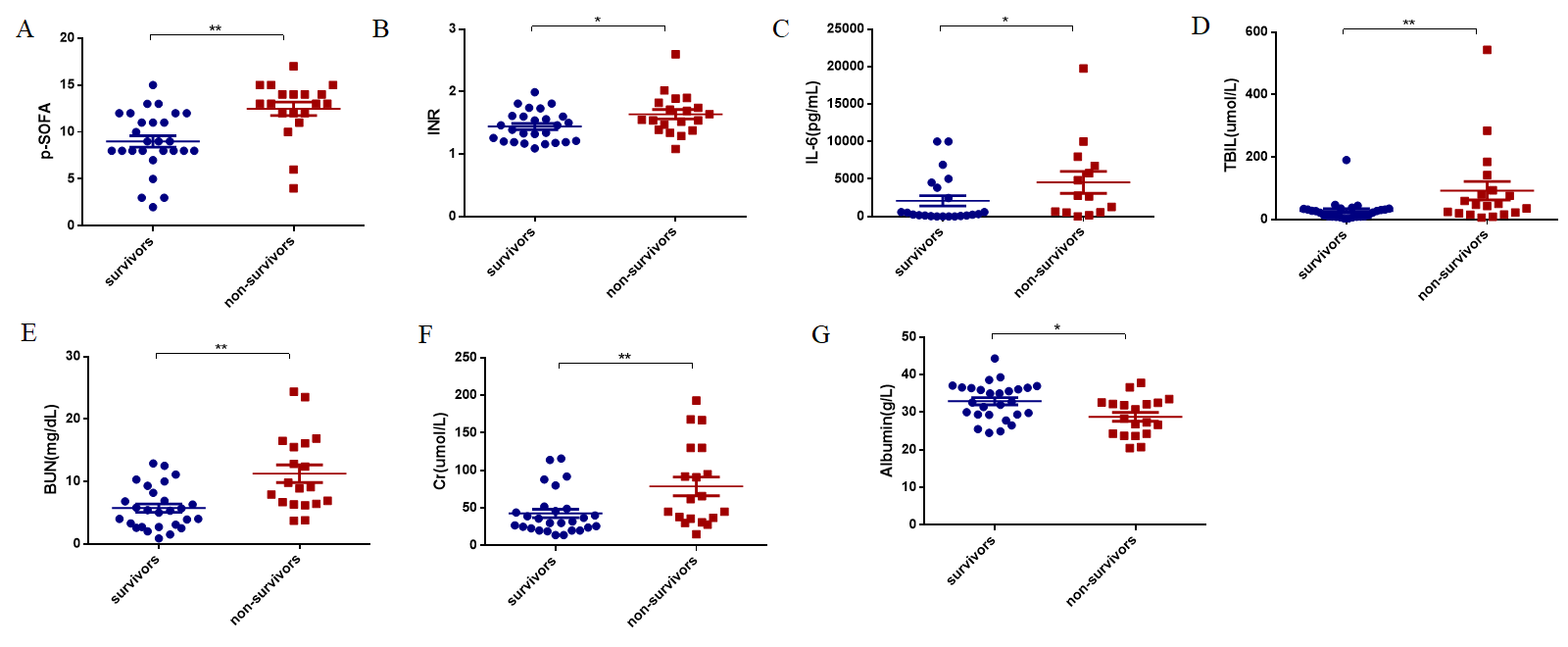


**Supplement 2.** The levels of p-SOFA scores, INR, IL-6, TBIL, BUN, sCr, and albumin in cancer-related sepsis group between survivor and non-survivors at CRRT initiation. (A) p-SOFA scores; (B) INR; (C) serum IL-6 levels; (D) serum TBIL levels; (E) serum BUN levels; (F)serum Cr levels; (G) serum albumin levels.

**Supplement 3.** Multivariable Logistic Regression Analysis for PICU

| variable | β(SE) | Wald | Degrees of Freedom | *Odds Ratio* (95%*CI*) | *P* value |
| --- | --- | --- | --- | --- | --- |
| p-SOFA | 0.278 | 4.516 | 1 | 1.805(1.047,3.113) | 0.034* |
| Albumin | 0.136 | 4.186 | 1 | 0.758(0.581,0.988) | 0.041* |
| TBIL | 0.011 | 0.053 | 1 | 1.002(0.982.1.024) | 0.818 |
| IL-6 | 0.001 | 0.161 | 1 | 1.000(1.000,1.000) | 0.689 |
